# The Use of Audiovisual Distraction Tools in the Dental Setting for Pediatric Subjects with Special Healthcare Needs: A Review and Proposal of a Multi-Session Model for Behavioral Management

**DOI:** 10.3390/children11091077

**Published:** 2024-09-02

**Authors:** Massimo Pisano, Alessia Bramanti, Giuseppina De Benedetto, Carmen Martin Carreras-Presas, Federica Di Spirito

**Affiliations:** 1 Department of Medicine, Surgery and Dentistry “Scuola Medica Salernitana”, University of Salerno, Via Salvador Allende, 84081 Baronissi, Italy; abramanti@unisa.it (A.B.); fdispirito@unisa.it (F.D.S.); 2 Oral Medicine Unit, Department of Dentistry, Faculty of Biomedical and Health Sciences, Universidad Europea de Madrid, 28670 Madrid, Spain; carmen.martin2@universidadeuropea.es

**Keywords:** pediatric dentistry, special health care needs, audio-visual tools, teledentistry, behavioural management

## Abstract

Background: A Special Health Care Need (SHCN) is characterized by any type of physical, mental, sensorial, cognitive, emotional, or developmental condition that requires medical treatment, specialized services, or healthcare interventions. These conditions can negatively impact oral health as SHCN children can hardly cooperate or communicate and experience higher levels of dental fear/anxiety, which interfere with regular appointments. The present narrative review aims to analyze the use of audiovisual (AV) tools in dental setting for the management of SHCN children during dental treatment and to evaluate their effectiveness in anxiety/behavior control from the child, dentist, and care-giver perspectives. This analysis leads to the proposal of a new multi-session model for the behavioral management of SHCN pediatric subjects. Methods: An electronic search on the MEDLINE/Pubmed, Scopus, and Web of Science databases was carried out and through this analysis, a new model was proposed, the “UNISA-Virtual Stepwise Distraction model”, a multi-session workflow combining traditional behavior management and the progressive introduction of AV media to familiarize the SHCN child with dental setting and manage behavior. Results: AV tools helped in most cases to manage SHCN behavior and decreased stress in both the dentist and child during dental treatments. Care-givers also welcomed AV distractors, reporting positive feedback in using them during future treatments. Conclusions: The present narrative review found increasing evidence of the use of AV media for SHCN pediatric subjects as distraction tools during dental treatment. In the majority of the studies, AV tools proved to be effective for the management of anxiety, dental fear, and behavior in dental setting.

## 1. Introduction

Special Health Care Needs (SHCNs), according to the American Academy of Pediatric Dentistry, are characterized by any type of physical, mental, sensorial, cognitive, emotional, or developmental condition that requires medical treatment, specialized services, or specific healthcare interventions. Such impairments could be congenital, acquired due to illness or injury, and may impact children’s and care-givers’ quality of life [1]. To provide healthcare for subjects with SHCNs, specific expertise, awareness, and attention, as well as tailored adaptation, are required [1].

SHCNs thus include subjects with congenital conditions such as trisomy 21 or heart malformations, developmental conditions such as cerebral palsy or cognitive and intellectual disabilities, and systemic diseases or genetic syndromes, along with behavioral conditions such as anxiety, attention deficit and hyperactivity disorder (ADHD), or autism spectrum disorder (ASD) [2]. These conditions can negatively impact oral health, particularly resulting in poorer oral hygiene and an increased risk of caries, fractures, erosions, bruxism, periodontitis, and malocclusions [3,4].

In addition, behavioral issues represent an extremely challenging barrier for dentists to overcome [5] as pediatric SHCN patients can hardly cooperate or communicate effectively and often have higher levels of dental fear and anxiety, which has a negative influence on regular appointments and treatments [6].

Several approaches have been proposed for the behavior management of pediatric subjects, which could be divided into basic or advanced techniques [7]. Basic behavior management methods include tell–show–do (TSD), positive reinforcement, rewards and modeling, non-verbal communication, and distractions. Advanced techniques involve protective stabilization, sedation, and general anesthesia [7].

Alongside traditional behavioral management techniques, distraction techniques based on auditory or audiovisual (AV) distraction tools, through the use of tablets, televisions, and projections, are becoming increasingly popular nowadays [8,9]. With the development of technology, the use of virtual reality (VR) has also been proposed in the dental environment to be able to manage children’s behavior during treatment [10,11]. Specifically, VR allows the creation of a three-dimensional (3D) model, which gives the user the ability to interface with a realistic 3D world in his eyes and, by the use of goggles or visors, enables the user to cover the view of the surroundings [12]. Another type of AV distraction can be provided through the use of AV goggles that arrange for the conversion of multimedia content, allowing the user to be immersed in a VR experience that is comparable to those of movies or the theater [10].

As SHCN individuals often experience difficulty in interfacing with the dental setting, the development of technologies has allowed the opportunity to explore new methods for managing children’s behavior and anxiety regarding dental treatments.

The aim of the present narrative review is, therefore, to analyze the use of AV tools in the dental setting for the management of pediatric SHCN subjects during dental treatment and to evaluate their effectiveness in managing anxiety and behavior at the child level and perspectives at the dentist and care-giver levels. This analysis leads to the proposal of a new multi-session model for the behavioral management of pediatric patients with special needs.

## 2. Materials and Methods

### 2.1. Search Strategy and Eligibility Criteria

An electronic search on MEDLINE/Pubmed, Scopus, and Web of Science databases was carried out by two independent reviewers (M.P.; G.D.B.) until 22 April 2024 using the following keywords combined with Boolean operators:

1. (“virtual reality” OR eyewear OR “audiovisual distraction” OR “augmented reality” OR video*)

AND

2. (“special need*” OR autism* OR “down syndrome*” OR deaf OR “impaired children” OR disability*)

AND

3. (dentistry OR “dental setting” OR pedodontics OR “dental treatment” OR “dental therapy” OR “dental therapies”)

An additional manual search through screening of the references of the included studies was performed.

Inclusion criteria were as follows:

- Source: Studies in the English language with no date restriction published until 22 April 2024.
- Design: Prospective, retrospective, and case–control studies; case series; case reports; randomized controlled trials; letters to editors.
- Population: Pediatric subjects with special healthcare needs without gender or age restrictions.
- Intervention: Use of audiovisual aids in the dental setting concerning dental therapy or prophylaxis.
- Outcomes: Reported outcomes of audiovisual aid intervention(s).

Exclusion criteria were as follows:

- Source: Studies not published in the English language.
- Design: In vitro studies, pre-clinical in vivo studies, reviews, conference papers, commentaries, book chapters.
- Population: Adult subjects or pediatric subjects without special healthcare needs.
- Intervention: Non-use of audiovisual aids or use of audiovisual aids not in dental setting or remotely delivered.
- Outcomes: No reported outcomes of audiovisual aid intervention(s).

Study selection was performed by three independent reviewers (A.B.; C.M.C.-P.; F.D.S.) and after the elimination of duplicates, titles and abstracts obtained from the search were analyzed. Full texts were obtained for potentially eligible studies, and if they were not available, the study authors were contacted.

References were collected and organized through Mendeley Reference Manager Software, version 2.120.3, copyright Elsevier Ltd.

### 2.2. Data Extraction and Collection

Data extraction and collection were carried out by two independent reviewers (F.D.S., A.B.) in a standardized form for studies included in the present narrative review. In the event of any disagreement, the reviewers resolved through discussion and by including a third reviewer if necessary (C.M.C.P.). For each record, the following data were collected:

Study: First author, year, study design;

Population: Number of participants, age, target population;

Methods: Aim; assessment tool;

Intervention: Intervention, dental therapy;

Outcome(s): Main result, child-level, dentist-level, care-giver level.

## 3. Results

### 3.1. Study Selection

A total of 298 records were obtained—precisely, 115 from MEDLINE/Pubmed, 146 from Web of Science, and 37 from Scopus—and 94 duplicates were removed. Of the 204 remaining records, through the screening of titles and abstracts, 146 were excluded as not relevant, and of the remaining records, 58 full texts were evaluated. Of these, forty-eight were excluded, specifically because ten were reviews, five involved adult subjects, two did not concern pediatric subjects with special healthcare needs, two did not mention whether the population was pediatric or adult, six did not report the use of audiovisual means, two were not in a dental setting, and twenty-one involved remotely delivered means.

**Figure 1:**
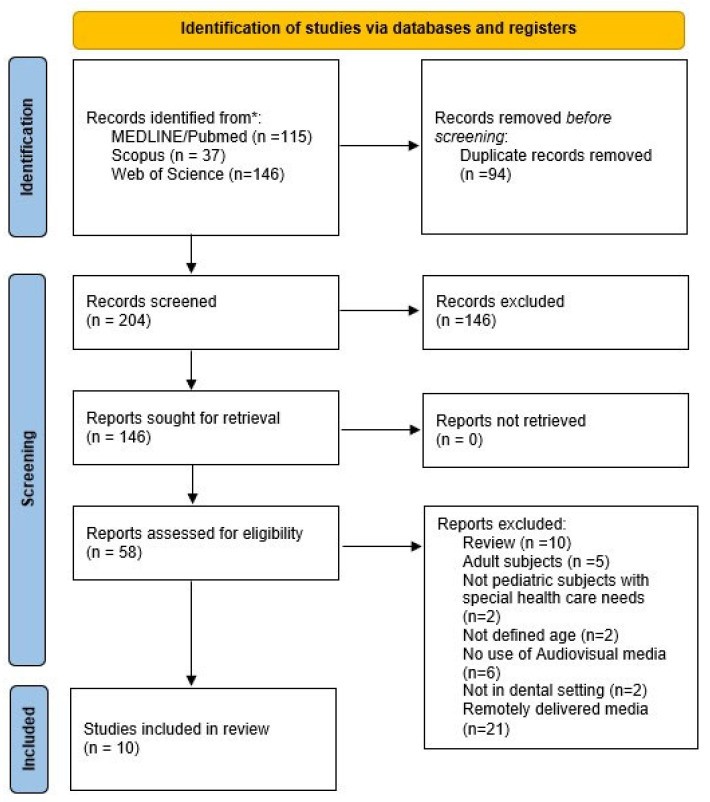
Study selection flowchart.

Figure 1 shows the flowchart of study selection.

After the screening of the reference lists of the articles included from the electronic search, one additional article was included by manual search in this narrative review [13].

### 3.2. Data Collection

The data extracted and collected from the included studies [13,14,15,16,17,18,19,20,21,22,23] in the present review are shown in Table 1.

### 3.3. Study Characteristics

The present review included eleven studies [13,14,15,16,17,18,19,20,21,22,23], of which three were randomized controlled trials [15,16,22], three were randomized crossover clinical trials [18,20,21], four were clinical studies [14,19,21,23], and one was a clinical trial [13].

The population examined in the included studies amounted to 424 pediatric subjects with special healthcare needs, in particular Down syndrome (n = 70) [15,19], ASD (n = 176) [21,22,23], ADHD (n = 31) [13], hearing impairment (n = 55) [16,17], hearing and speech impairment (n = 24) [14], intellectual disabilities (n = 20) [18], genetic syndromes, congenital heart diseases, cancer, and organ transplants (n = 48) [20]. The age of the population ranged from 5 to 17 years old.

The majority of the studies compared the use of AV distraction media with another management technique during a dental procedure such as oral examination, professional oral hygiene, fluoride or sealing applications [13,14,16,21,22,23], and restorative dental treatment [15,17,18,19,20]. For some studies, dental procedures included multiple sessions with several treatments performed [13,17,18,19,21,22,23].

The AV media used in each study was also investigated. In particular, seven studies used AV eyeglasses [13,15,17,19,20,21,22] while four chose VR eyeglasses [14,16,18,23]. The comparison with other techniques included AV projection [13,17,19,21], video peer modeling [22], visual distraction via a tablet [14], audio distraction with headphones [18], video peer modeling, using AV glasses [22], conventional behavior management techniques [15,23], conventional behavior management, protective glasses [20], and tell–show–do or modified tell–show–do [13,16,19] or using no AV distractions [14,22].

### 3.4. Anxiety, Pain, and Behavior Assessment Tools and Scales

In the included studies, several scales and tools were used to assess anxiety, pain, and children’s behavior. In particular, the reported anxiety scales were Vehnham’s Anxiety Rating Scale [18], which consists of scores ranging from 0 to 5, thus varying from no or slight levels of anxiety to clearly anxious and avoidant behavior toward the dentist [24], and the facial image scale (FIS) [16], which uses pictures to evaluate anxiety in children [25]. Some studies referred objective physiological parameters, namely oxygen saturation [13,17,18,19,21], the pulse rate [13,14,16,17,18,19,21,22], and blood pressure [14], to assess anxiety.

The behavior scales included in the studies were Frankl’s scale [15,23], which is based on four categories (1–4) ranging from definitively negative to definitively positive behavior [26]; Venham’s Behaviour Rating Scale [23], which is a six-point scale (0–6) ranging from fully cooperative behavior to behavior that is not cooperative [26]; VAS [15,20], consisting of a horizontal line whose extremes represent negative and positive behavior [26]; and the pictorial scale [14].

The pain scales used in the included studies were the Revised Face, Legs, Activity, Cry, Consolability (r-FLACC) scale [15,20], which is a scale based on three categories (0–3) assessing the face, the legs, activity, crying, and consolability [27]; Revised Faces Pain Scale (FPS-R) [20], which assesses pain through an image-based scale depicting faces [28]; and Wong–Baker’s Faces Pain Scale [17], which is a scale based on 10 levels of severity, ranging from 0 to indicate “no hurt” to 10 to indicate “hurt worst” [29].

### 3.5. Dental Treatment

The data extracted from the included studies of this narrative review could be organized, summarized, and described by the type of dental treatment performed.

#### 3.5.1. Oral Examination, Professional Oral Hygiene, Fluoride Applications, and Dental Sealing Placement

Nine studies [13,14,16,17,18,19,21,22,23] reported several dental treatments, in particular oral examination [18]; oral examination and professional oral hygiene [13,21]; dental impressions and professional oral hygiene [17,19]; professional oral hygiene [14,16]; and oral examination, professional oral hygiene, fluoride application, and dental sealant placement [22,23]. Specifically, the interventions included comparisons between AV eyeglasses and AV projection on subjects with ASD [13,21], ADHD [13], Down syndrome [19], hearing impairment [17]; groups with AV eyeglasses, video peer modeling and AV eyeglasses and groups with video peer modeling containing ASD subjects [22]; VR eyeglasses, visual distraction through a tablet, and no visual distraction in speech and hearing-impaired subjects [14]; VR eyeglasses and audio distraction in intellectually impaired subjects [18]; and a group with VR eyeglasses with a modified TSD technique and a group with a modified TSD technique in subjects with hearing impairment [16].

The majority of studies that compared the use of AV eyeglasses with AV projection showed the efficacy of using AV eyeglasses in anxiety control and behavior management in individuals with ASD, down syndrome, and ADHD [13,19,21]. In addition, at the dentist level, the use of glasses and multiple visits increased compliance in subjects with ADHD [13]. In subjects with hearing impairment, the use of AV projection was more recommended than the use of AV eyeglasses [17].

The use of AV eyeglasses alone or in combination with video peer modeling has shown effectiveness in controlling the behavior and anxiety of ASD subjects when compared to peer modeling alone [22].

In the reviewed studies, the employment of VR eyeglasses was more effective in reducing anxiety than visual distraction through a tablet or with no AV distraction during professional oral hygiene procedures in children with speech and hearing impairment [14].

The use of VR eyeglasses and audio distraction through headphones was found to be effective in both reducing anxiety and controlling the behavior of children with mild intellectual disabilities [18]. In addition, at the dentist level, the use of distractors was associated with a change in positive body language, and on the care-giver level, the use of distraction means was well welcomed and received positive feedback regarding their use in future treatments [18]. VR glasses in combination with a modified TSD technique showed higher effectiveness in reducing dental anxiety compared to the modified TSD technique alone in children with hearing impairment [16].

#### 3.5.2. Restorative Dental Treatment

Six studies [15,17,18,19,20,23] examined the use of AV media during restorative dental treatments. Specifically, two studies [15,20] compared the use of AV eyeglasses with conventional behavioral management techniques, two [17,19] compared the use of AV eyeglasses with AV projection, one [18] compared the use of VR eyeglasses with audio distraction through headphones, and one [18] compared the use of VR eyeglasses and conventional behavior techniques [23]. Among these, two studies also carried out other forms of dental care including oral examination, professional oral hygiene procedures, topical fluoride application, and sealant placement [18,23]. From the comparison of AV eyeglasses and conventional behavior management in the study carried out with Down syndrome subjects as the target population [15], eyeglasses generated less cooperation in the children and 64% refused to wear them. Furthermore, the r-FLACC scores and Frankl’s scale values were better when using conventional behavior management techniques. Concerning the dentist level, the operators showed similar VAS values for the two groups. In contrast, in the study performed on subjects with genetic syndromes, congenital heart diseases, cancer, and organ transplants [20], the use of AV eyeglasses was effective in controlling and managing children’s anxiety and behavior compared with conventional techniques, with lower r-FLACC and FPS-R values. In addition, children showed a preference for wearing glasses and higher satisfaction. On the dentist level, practitioners showed a preference for the use of AV eyeglasses during therapy, with significantly lower VAS values in than the conventional behavior technique group.

A comparison of AV eyeglasses and AV projections was performed during stainless crown placement in a Down syndrome population [19] and for root canal therapy in subjects with hearing impairment [17]. It was found that the use of AV eyeglasses was effective in managing children’s behavior and controlling anxiety in subjects with Down syndrome, who showed significant differences in the pulse rate during tooth preparation and anesthesia administration; on the dentist level, the use of eyeglasses also helped in covering the sound of the drills during the procedure. Among the population with hearing impairment, conversely, AV projection was more effective and showed a better pulse rate and self-reported pain scale values during endodontic therapy.

Studies comparing VR eyeglasses with conventional behavioral management techniques and the use of audio distraction through headphones were performed on subjects with ASD [23] and intellectual disabilities [18], respectively. In particular, in ASD children, the use of VR eyeglasses has been shown to be useful in behavioral control. Also, on the dentist level, it has allowed a reduction in the number of interruptions during treatment [23].

The comparison of VR eyeglasses and audio distraction in subjects with mild disabilities showed the efficacy of both means, resulting in a reduction in anxiety [18]. At the dentist level, the introduction of the distraction means resulted in a positive change in the children’s body language; furthermore, at the care-giver level, the authors found positive feedback and attitudes toward using AV media in future dental treatments [18].

**Figure 2:**
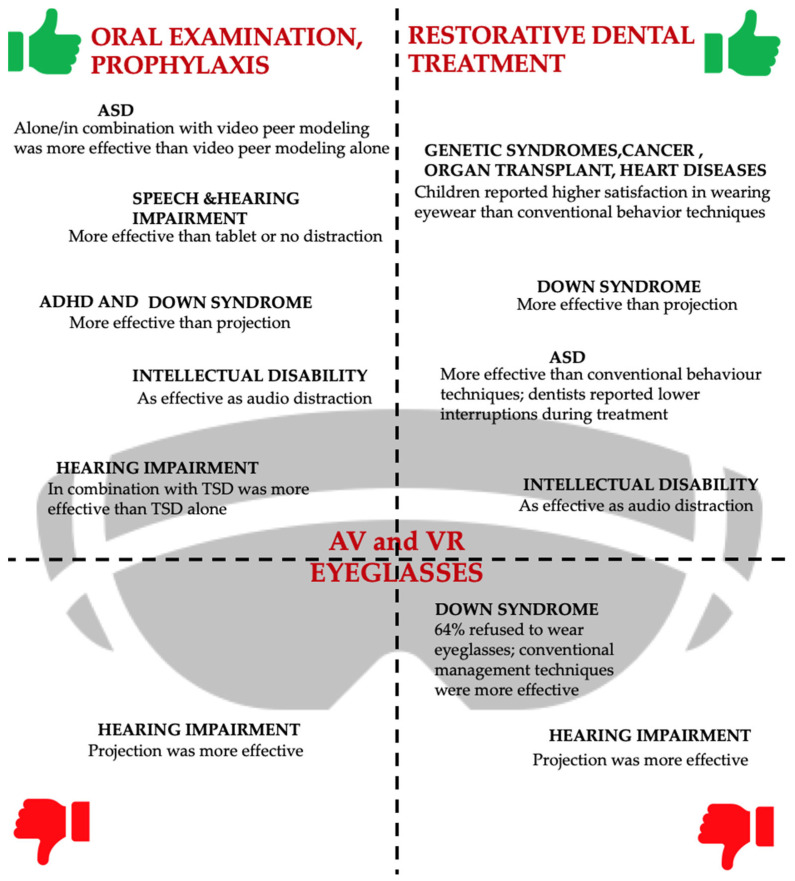
The effectiveness of the use of AV and VR eyeglasses during oral examination and prophylaxis (**left**) and restorative dental treatment (**right**) in relation to the SHCN target population. Abbreviations: Audiovisual—“AV”; virtual reality—“VR”; autism spectrum disorder—“ASD”; tell–show–do—“TSD”.

Figure 2 shows the effectiveness of the use of AV and VR glasses in relation to the type of dental treatment performed and by target population.

## 4. Discussion

Dental treatment in pediatric SHCN subjects may be particularly challenging because of communicational, behavioral, and psychosocial barriers that may arise between the dentist and the child, and often the care-giver as well, which may result in the avoidance of the dental environment, adversely affecting the child’s oral and systemic health [1,30]. Indeed, SHCN individuals may experience higher levels of dental anxiety compared to non-SHCN subjects, which could lead to aversion and resistance and, at times, even impulsivity or aggressive attitude toward the dental setting, making behavioral management difficult during dental treatment [1,6]. Some evidence suggests that the over-reliance of the dental treatment of SHCN subjects on deep sedation and general anesthesia by dentists [31,32], due likely to the ineffective scheduling of behavioral management strategies [33], especially because of the fact that such individuals may have sensory issues, intellectual disabilities, or impairments in speech and hearing [1], is a problem. The role of the dentist, therefore, may be a detriment in implementing effective behavioral management techniques in treating pediatric SHCN subjects to be able to create an environment of mutual cooperation and compliance between the child, care-giver, and dentist. Alongside conventional behavioral management techniques, which include TSD techniques, positive reinforcement, reward, distractions, and modeling [7], auditory distractions; AV distractions through the use of tablets, television, or projections [8,9]; and technologies such as VR or AV tools through goggles or visors could also play a role [10,11]. Therefore, the present review aims to analyze the use of AV tools in SHCN pediatric subjects and to evaluate their effectiveness in behavior management during dental treatment and assess the perspectives towards them at the child level, dentist level, and care-giver level.

### 4.1. Child Level

On the child level, based on the results obtained from this narrative review, behavior, anxiety, and dental fear management through the use of AV tools were shown to be heterogeneous to both the type of SHCN, among the same type of target population, as well as towards different preventive or curative dental treatments. In particular, the included study by Fakhruddin et al. [17] reported that during dental prophylactic cleaning, carried out on subjects with hearing impairment, the use of visual distraction through projection was shown to be more effective than the use of visual distraction through eyewear. This may be attributable to the fact that the use of eyewear may excessively isolate the subject and could generate a state of sudden fear if touched by the operator in the absence of eye contact, which is instead assured with full visibility by projection. Conversely, the study by Sanguida et al. [14] found the use of VR eyeglasses to be more effective in reducing anxiety, which could be explained by the fact that the target populations of the two studies, although the same, differed in the age range and, therefore, the use of glasses or visors might be more accepted in older pediatric subjects. In the same target population, a further study, included in the present narrative review [16], reported that the use of VR glasses helped in the controlling of dental fear and anxiety and that decreases in the pulse rate and FIS scores were found compared to the group managed with the TSD technique alone. The effectiveness of VR in the hearing impairment population may have been due to the combination of the TSD method, which is a procedure that helps the child become familiar with the dental setting and to avert fear of the unknown by describing what he is going to face [8], with AV distraction methods during dental treatment.

Among children with Down syndrome, Bagattoni et al. [15] found that the use of AV eyeglasses generated a lack of cooperation during restorative dental treatment and that high percentages of them refused to wear the devices, making traditional behavioral management techniques more effective.

In contrast, the efficacy of AV eyeglasses for the same target pediatric population was also suggested by Fakhruddin et al. during the placement of stainless crowns [19]. However, such different results may have been attributable to the difference in treatment planning. In fact, in the study where the use AV glasses was proven to be effective [19], the treatment was deferred over several sessions, which might have benefited the management of children more. Indeed, when treating pediatric SHCN subjects, consideration should be given not only to the lengths of appointments but also to the possibility that introductory visits or taking multiple split sessions may help the child become familiar with the dental setting and help guide him toward a more positive experience [2].

Moreover, based on the results found in the present review, the use of VR or AV eyeglasses as a distraction tool during visits and restorative or preventive treatments was found to be effective in behavioral management and anxiety control in pediatric individuals with ASD or ADHD. In most cases, the distractor tools were combined with other behavioral management strategies, such as TSDs, introductory visits, and video peer modeling that may have helped a child become more comfortable with the dental environment. Indeed, these children may be particularly sensitive to noise and have sensory characteristics that can make them particularly frustrated or easily distracted during treatment [6,13]. The use of AV goggles or visors or VR may have helped in covering the sounds of drills and desensitization to auditory and tactile stimuli. Indeed, to avoid the use of drills, which could trigger anxiety and discomfort in the SHCN individual, an alternative during conservative treatment could be the use of ozone [34]. Some studies suggest that its use is effective in treating caries in uncollaborative children with dental fear without undermining the quality of the restoration [35]. Ozone, in addition to its properties of being able to destroy caries bacteria and remove carious lesions, also has analgesic effects [36,37]. This may be important in the perception of pain, which may be found to be increased in SHCN subjects [38,39].

### 4.2. Dentist Level

The dentist holds a key role in providing well-rounded care and making the dental experience positive to promote good oral health in SHCN individuals [40]. Previous studies reported that SHCN subjects have poorer oral health and that inequalities exist compared with non-SHCN subjects [41,42]. Moreover, compared with the general population, the prevalence of untreated caries and periodontitis is higher [43]. This could be attributable to the lack of knowledge by dentists in managing SHCN subjects, children’s anxiety and avoidance of dental setting, and the concomitance of other systemic diseases that put oral health on the back burner by families [44,45].

Oral health has an important impact on overall health [40,46], so the proper management of pediatric SHCN individuals is required and oral care providers should have specific knowledge and training; dental setting should be adapted so that children achieve a positive attitude in the dental environment [45,47,48].

**Figure 3:**
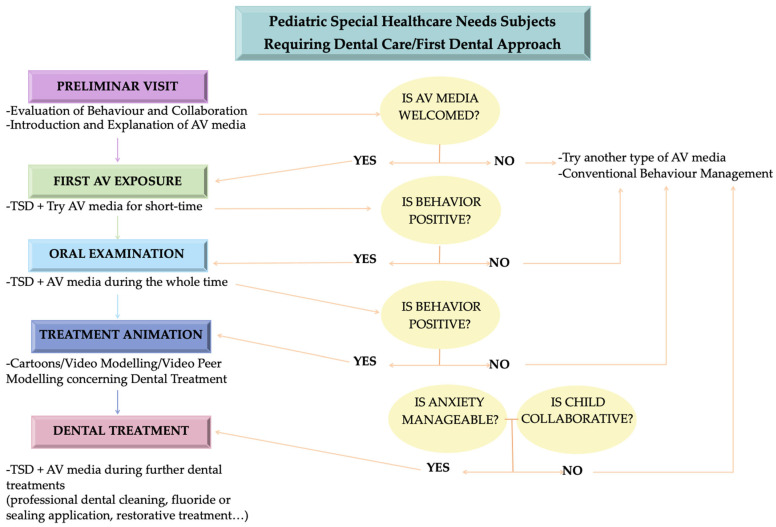
The “UNISA-Virtual Stepwise Distraction model”, a multi-session work-flow combining traditional behavior management and the progressive introduction of AV media to familiarize the SHCN child with the dental setting and manage behavior and anxiety. Abbreviations: Audiovisual “AV”; tell–show–do—“TSD”.

Based on the results of the present review, the use of AV distractor means helped in most cases to manage the behavior of SHCN subjects and generated less stress in both the operator and the child during dental treatments. The greatest effectiveness of the use of AV goggles or eyeglasses and VR occurred when they were combined with multiple visits or other behavior management techniques and educational or distraction methods [13,16,22,23]. Therefore, the authors have proposed a working model, the “UNISA-Virtual Stepwise Distraction Model”, that may be useful in introducing the SHCN child to the dental setting and help cope with anxiety toward dental treatment through the use of AV means so that the dentist can manage the behavior properly. Figure 3 shows the suggested work-flow, named the “UNISA-Virtual Stepwise Distraction Model”.

As reported by some evidence and findings in this narrative review, deferring visits over several sessions is effective in creating a positive attitude and in familiarizing the child with the dental environment [2,13,45]. The authors suggest, then, that treatment begin with a preliminary visit, which aims to desensitize the subject to the dental environment, for the initial assessment of behavior and cooperation and for the introduction and explanation of the AV medium, such as eyewear or visors, to be used. When the child accepts the AV medium willingly, the first AV exposure could begin, in which the child tries out the AV medium of choice for a short period of time and through which the subject can watch a cartoon or a short animated video. After the exposure testing, the dentist can assess the child’s behavior. Behavior can be assessed using different scales such as Frankl’s scale, Vehnam’s scale, VAS, or the pictorial scale [20,26]. If the behavior is negative, the dentist may proceed with trying other means of distraction such as AV projection or conventional behavioral management techniques.

If, on the other hand, the attitude towards the AV medium is positive, the next session will be characterized by the first visit with the oral examination, which will first be introduced through the TSD method, then showing the tools that will be used such as the mirror or probe, then conducted using the AV medium of choice as a distraction during the visit. If the dentist has assessed positive behavior, the next session can proceed with the treatment the child needs. Some evidence has reported the effectiveness of online or remotely delivered dental educational videos [49,50,51]. If the content view, showing the treatment to be performed through cartoons or video peer modeling, results in cooperation and manageable dental fear and anxiety, the child can proceed with the dental treatment, introduced before the session through the TSD technique and performed with the AV medium of choice in support. The “UNISA-Virtual Stepwise distraction Model”, a combined multi-session work-flow with traditional behavioral management methods and AV distraction means, may be useful in getting the child to enthusiastically approach the dental environment or to manage children who have had negative dental experiences in the past.

Also, on the dentist level, an aspect to consider is the role dentists play in the prevention and promotion of oral health among children and care-givers [45]. Indeed, evidence suggests that the majority of SHCN individuals require more invasive dental treatment [52], which may suggest the need for prevention programs to bridge the inequality gap in the oral health status compared to the non-SHCN population.

Therefore, the dentist plays a role in dental education by teaching and motivating the child in oral hygiene and healthy diet maintenance, by means of using a targeted approach to the child’s communication skills, and also by educating the care-giver and families towards greater awareness of the importance of dental care and regular check-ups that impact the overall health of the child [53].

### 4.3. Care-Giver Level

Care-givers of SHCN subjects may be subjected to higher levels of stress and frustration due to the often difficult behavioral management of children, especially in new environments such as dental settings [54,55].

In addition, some evidence suggests that among care-givers of SHCN individuals, social support is poorer due to stigma and that this may affect less access to dental care and dental knowledge [56,57,58].

Some studies focusing on parents’ perceptions about dental experiences have complained about a lack of interest from dentists in how to proceed with their children and whether there were sensory difficulties to certain stimuli present in the dental environment, such as lights or noises such as those of drills [59].

In addition, some care-givers showed that interest in oral health was low because of the stress it entails for the child and the frustration it induces in the parents themselves [59,60].

Indeed, it is important that the care-giver, along with the pediatric patient, are satisfied with the dental services received, given the increasing attention to and accessibility of information related to dental care [61]. This aspect takes on additional relevance where pediatric SHCN subjects are concerned as care-givers’ satisfaction becomes a crucial factor in healthcare delivery [9]. Satisfied parents/care-givers are more likely to ensure that their children follow good oral hygiene habits and consistently undergo dental examinations, thus leading to a possibility of improvement in the child’s oral health, as an attempt to break down inequality and disparities [62].

Evidence suggests that care-giver support through the use of tools before and during visits together with desensitization visits helps the SHCN child experience the dental environment [63,64].

According to the results of this review, care-givers welcomed the use of AV distraction tools during visits and treatments and reported positive feedback in using these tools to help children accept dental care in the future. These findings are in line with previous studies that showed that parents welcomed the use of other AV media distractors through the use of tablets, computers, projection, and social stories in controlling behavior and anxiety [65,66]. Whenever the child is particularly reluctant to the dental environment, parental support may also be provided through teledentistry [67]. Indeed, it was particularly helpful during the COVID-19 pandemic in being able to ensure equitable access to care [68,69]. Teledentistry may be useful in monitoring oral health and limiting the cost and time of visits [70], which could be particularly useful for care-givers who have difficulty interfacing with appropriate specialists for SHCN children or have socioeconomic difficulties [30,71]. In addition, it might be an integral part of the establishment of a dental home that has proven to be useful in the care of SHCN children and parents and the interfacing between specialists in providing all-round care for the SHCN pediatric subject, which is also useful to ensure a relationship of mutual trust [72,73]. Moreover, support through such resources can not only have an impact on the health of SHCN individuals but also reflects on that of care-givers as well [54]. Support through these tools increases awareness in care-givers as they allow the opportunity for easier access to information related to dental education and health and increase resources that facilitate SHCN children’s dental visits and limit inequalities compared to non-SHCN individuals [30]. Indeed, a positive vicious circle might be established through access to such information and resources: while care-givers have an impact on the oral health status of children, an indirect mechanism might exist whereby access to such services directed at the SHCN child might determine enhance the oral health of care-givers as well.

### 4.4. Future Perspectives and Applications

As technology development advances, new perspectives in the field of medicine and dentistry regarding artificial intelligence (AI) have shown to be promising in pedodontics to increase oral health, diagnostic accuracy, and effectiveness and provide customized dental care [74]. AI has shown its potential in the evaluation of dental plaque, identification of supernumerary teeth, and detection of early childhood caries [75]. Wang et al. [76] developed a toolkit based on machine learning to be able to assess a child’s oral health status and need for treatment and to help and guide the dentist in in-person oral examination. Such AI-based toolkits might be useful for pediatric SHCN subjects, both on the dentist level to get a preliminary idea about oral care needs and also on the care-giver level to increase awareness of the importance of oral health.

The progress of computer-based technologies alongside VR also includes augmented reality (AR) [12], which creates virtual images by placing them in the real world so that the subject sees a real environment enhanced by digitized graphics and details [77]. AR-based training helps the subjects participate actively in tasks while at the same time enjoying and increasing their skills in the required tasks through game content activities [78]. In addition, AR tools have sensors capable of recognizing movement that allow the results of the user’s activities to be monitored immediately [79]. AR-based training has been applied in dentistry to improve tooth-brushing skills [80]. SHCN children often avoid toothbrushing due to sensory issues with the taste or texture of toothpaste or the pressure applied by the toothbrush [40]. Zengh et al. [81] proposed an AR educational system, “CheerBrush”, catering to ASD subjects. This technology aims to help the children handle a virtual toothbrush and toothpaste and practice brushing movements while sensors detect motions and give back results [81]. Furthermore, a technological model for the management of pediatric ASD subjects, based on the use of AR, has been proposed by Pagano et al. [82] to increase collaboration between the child, dentist, and care-giver. This technology is based on a virtual immersion of the child together with the dental care provider and the care-giver in the dental setting and visualization of the future dental treatment that will be performed so as to increase consciousness and promote better behavioral management in the dental environment [82]. Further studies are needed to highlight the potential of such promising technologies among pediatric SHCN subjects in the dental setting as educational tools and as adjunct aids during treatments.

On the dentist level, the use of technologies might be useful to fill the lack of knowledge about SHCN population management through the uploading of virtual patient models. Kleinert et al. proposed a full-screen virtual model, intended for dental students, in which a simulation of an oral examination of a child with Down syndrome was uploaded to assess whether such a model increased knowledge in the care and management of pediatric SHCN subjects [83].

With technological advancement, such virtual patient models in dental settings might be present as VR or AR tools so that the dental care provider may be totally immersed in a “virtualized” real-world situation and be able to be monitored and guided toward the most appropriate and relevant choices for the SHCN population.

In the future, university dentistry courses might prospect the use of such technologies to provide adequate knowledge to students, the future dental care providers, in the appropriate management of pediatric SHCN subjects; to increase expertise in communication strategies and behavior control techniques; and to spread awareness of the need to overcome disparities.

Further studies may therefore be needed to be able to investigate the potential of tools using VR or AR to increase awareness at the dentist level.

## 5. Strength and Limitations

The limitation of the present review, as with narrative reviews, was the lack of a pre-defined systematic and rigorous methodology, which may have made it subject to biases. However, despite their limitations, narrative reviews offer the possibility of providing insight into a topic in such a way as to increase awareness and knowledge. Indeed, the purpose of this study was to describe the use of AV tools in the dental setting and evaluate their effectiveness in controlling behavior and anxiety at the child, care-giver, and dentist levels. In addition, based on our knowledge, the authors provided, for the first time, a working model to be able to manage the behavior of SHCN children during dental treatment with a multi-session approach based on a combination of conventional techniques and the gradual introduction of AV media.

## 6. Conclusions

The present narrative review found increasing evidence of the use of AV media for SHCN pediatric subjects as distraction tools during dental treatment. In the majority of the reviewed studies, AV tools proved to be effective for the management of anxiety, dental fear, and behavior in the dental setting. One aspect to be emphasized is that such AV tools, together with conventional management techniques, can help establish a collaborative climate between the dentist, child, and care-giver to achieve a positive approach in dental health education. With the development and technological advances, through VR, AV goggles and visors, AR, and AI, it is possible to glimpse into the future so that these technologies may enhance the behavioral management of SHCN children and modulate anxiety and avoidance towards the dental setting. Therefore, further studies may be necessary to extensively investigate the potential of such technologies and their application in dentistry for the SHCN pediatric population.

## Figures and Tables

**Table 1:** Data extraction and collection from the included studies according to the following parameters: study (first author, year, study design), population (number of participants, age, target population), methods (aim, assessment tool), intervention (intervention, dental therapy), and outcomes (main result, child-level, dentist-level, care-giver level).

Study	Population	Methods	Intervention	Outcome(s)
Bagattoni et al., 2017 Randomized Crossover Clinical Trial [20]	Nr. of participants: 48 Age: 5–10 years old Target Population: genetic syndromes, congenital heart diseases, cancer, organ transplant	Aim: To evaluate the effects of AV distraction on children’s behaviors, perception of pain, treatment duration, and operator stress. Assessment tools: FPS-R, r-FLACC, VAS	Intervention: AV eyeglasses (Group 1 nr. 24); Conventional behavior management + protective glasses (Group 2 nr. 24). The groups interchanged in the second session. Dental therapy: Restorative dental treatment.	Main result: AV distraction is effective in managing stress and anxiety in children and operator. Child-level: r-FLACC scores and FPS-R scores were significantly lower using the AV eyeglasses in the second session. A total of 83% of participants had more satisfaction in using AV eyeglasses. Dentist-level: Operator stress showed significantly lower VAS scores with AV eyeglasses group. Care-giver-level: M.D.
Bagattoni et al., 2020 Randomized controlled trial [15]	Nr. of participants: 48 Age: 5–12 years old Target Population: Down syndrome	Aim: To evaluate the effects of AV distraction on children’s behaviors, perception of pain, operator stress, and treatment duration. Assessment tool: r-FLACC, Frankl, VAS	Intervention: AV eyeglasses (Group 1 nr. 24); Conventional behavior management techniques (Group 2 nr. 24). Dental therapy: Restorative dental treatment with topical anesthesia.	Main result: AV eyeglasses created less cooperation during treatment compared to conventional management techniques. Child-level: A total of 64% refused to wear AV eyeglasses; r-FLACC scores were higher; 68% showed negative behavior (Frankl 2). Dentist-level: Operator stress showed similar VAS scores between groups. Care-giver-level: M.D.
Chaitanya et al., 2023 Randomized controlled trial [16]	Nr. of participants: 40 Age: 6–11 years old Target Population: hearing impairment	Aim: To assess the effects of VR and the modified TSD method on dental anxiety levels. Assessment tool: FIS, pulse rate	Intervention: VR glasses + modified TSD (Group 1 nr. 20); Modified TSD (Group 2 nr. 20). Dental therapy: professional oral hygiene.	Main result: The use of VR glasses in combination with modified TSD method is more effective in reducing dental anxiety in hearing-impaired children compared to the modified TSD method alone. Child-level: Decreases in pulse rate and FIS score were noted in Group 1 Dentist-level: M.D. Care-giver-level: M.D.
Fakhruddin et al., 2016 Randomized Crossover Clinical Trial [17]	Nr. of participants: 15 Age: 5–7 years old Target Population: hearing impairment	Aim: To assess the effects of visual distraction (with/without glasses) in anxiety levels and the pain scale. Assessment tool: Wong–Baker’s Faces Pain Scale, oxygen saturation, pulse rate	Intervention: AV glasses (Group 1 nr. 7); AV projection (Group 2 nr. 8). In session 1, both AV media were used (nr. 15). During sessions 2–3, the two groups interchanged media. Dental therapy: Session 1: professional oral hygiene and dental impressions; Session 2–3: pulp therapy.	Main result: The use of AV glasses with full visibility of the surroundings is advisable during dental treatment for effective behavior management in hearing-impaired children. Child-level: Between sessions 2 and 3, the pulse rate improved in the AV glasses group; the self-reported pain score showed a statistically significant increase for the AV glasses. Dentist-level: M.D. Care-giver-level: M.D.
Fakhruddin et al., 2017 Clinical study [21]	Nr. of participants: 28 Age: 6.5–9.8 years old Target Population: ASD	Aim: To evaluate the effectiveness of AV distraction in managing children’s behaviors. Assessment tool: oxygen saturation, pulse rate	Intervention: AV eyeglasses; AV projection. In session 1, both AV media were used; in sessions 2–3, AV eyeglasses were used. Dental therapy: Session 1: oral examination, X-rays; Session 2–3: professional oral hygiene; dental sealant placement.	Main result: AV eyeglasses are effective in managing ASD children during dental treatments. Child-level: During the use of AV glasses, a reduction in the pulse rate was found, which demonstrated a decrease in anxiety levels; no statistically significant differences were noted in oxygen saturation levels. Dentist-level: M.D. Care-giver-level: M.D.
Fakhruddin et al., 2017 Clinical study [19]	Nr. of participants: 22 Age: 6.2–9.5 years old Target Population: Down syndrome	Aim: To assess the effectiveness of AV distraction (with/without glasses) and TSD in combination with computerized delivery anesthesia during the placement of stainless steel crowns. Assessment tool: oxygen saturation, pulse rate	Intervention: AV eyeglasses; AV projection. In session 1, both AV media were used; in session 2–3, AV eyeglasses were used. Dental therapy: Session 1: professional oral hygiene; dental impressions; Session 2–3: Computer-delivered anesthesia, tooth preparation, and crown cementation.	Main result: The use of AV glasses may be recommended to better manage children with Down syndrome during dental treatment Child-level: During session 1, statistically significant difference in the pulse rate between the AV groups with/without glasses was noted; during sessions 2–3 an increase in the pulse rate during tooth preparation with drills and a decrease during the administration of computer-delivered anesthesia with AV glasses has been found; no statistically significant difference in oxygen saturation levels was noted. Dentist-level: The use of AV glasses helped cover the sound of the drills. Care-giver-level: M.D.
Fakhruddin et al., 2018 Clinical Trial [13]	Nr. of participants: 31 Age: 6.5–8.1 years old Target Population: attention deficit/hyperactivity disorder (nr. 17 no medication; nr. 14 on medication)	Aim: To assess the effectiveness of AV distraction (with/without glasses) during dental treatment. Assessment tool: oxygen saturation, pulse rate	Intervention: AV eyeglasses; AV projection. In sessions 1–4, both AV media were used. Dental therapy: Session 1: desensitization appointment with TSD method; Session 2: dental examination and dental charting; Session 3: professional oral hygiene; Session 4: dental sealant placement.	Main result: The use of AV glasses and multiple visits may be recommended to better manage children with attention deficit/hyperactivity disorder. Child-level: No significant difference in oxygen levels during sessions 1–3 was noted; significant differences in the pulse rate were noted during session 4 with AV glasses. Dentist-level: Splitting the treatments into multiple short visits is beneficial for better behavioral management; using session 1 as a desensitization appointment with TSD is helpful for obtaining compliance. Care-giver-level: M.D.
Isong et al., 2014 Randomized controlled trial [22]	Nr. of participants:80 Age: 7–17 years old Target Population: ASD	Aim: To assess the effectiveness of electronic media strategies in reducing fear and increasing children’s compliance. Assessment tool: Venham Anxiety Rating Scale, Venham Behaviour Rating Scale, pulse rate	Intervention: AV eyeglasses (Group 1 nr. 20); Video peer modelling (Group 2 nr. 20); Video peer modeling + AV glasses (Group 3 nr. 20); No AV distraction (Group 4 nr. 20). Dental therapy: Session 1: visit (extraoral and intraoral examination, scaling, prophylaxis, fluoride varnish application, radiographs); Session 2: follow-up visit (extraoral and intraoral examination, scaling, prophylaxis, fluoride varnish application, radiographs).	Main result: The use of electronic media may improve the fear and compliance in ASD children during dental visits. Child-level: Between sessions 1 and 2, anxiety and behavior scores improved significantly in the AV glasses and video peer modelling + AV glasses groups; behavior scores were lower in the video peer modelling + AV glasses group; no statistically significant difference was noted among groups in the pulse rate. Dentist-level: M.D. Care-giver-level: M.D.
Mehrotra et al., 2023 Randomized Crossover Clinical Trial [18]	Nr. of participants: 20 Age: 6–14 years old Target Population: mild intellectual disability	Aim: To evaluate and compare audio and VR distraction tools on dental anxiety levels. Assessment tool: Vehnham’s Anxiety Rating Scale, pulse rate, and oxygen saturation.	Intervention: VR eyeglasses (nr. 10); audio distraction with headphones (nr. 10). Between sessions 1 and 2, the two groups interchanged. Dental therapy: Session 1: intraoral examination and restorative dental treatment; Session 2: intraoral examination and restorative treatment.	Main result: VR and audio distraction are both effective in reducing anxiety and managing children’s behaviors. Child-level: No statistically significance difference between VR and audio was found. The introduction of the distraction tool involved decreases in the pulse rate and anxiety levels and an increase in oxygen levels. Dentist-level: With the introduction of the distraction tool, a positive change in the participant’s body language was found. Care-giver-level: Positive feedback was received from parents during the treatment and on the use of distraction tools in future appointments.
Sanguida et al., 2021 Clinical study [14]	Nr. of participants: 24 Age: 6–12 years old Target Population: speech hearing impairment	Aim: To assess the effectiveness of visual distraction (with/without VR glasses) in anxiety levels. Assessment tool: pictorial Scale, pulse rate, blood pressure.	Intervention: VR eyeglasses (Group 1 nr. 8); Visual distraction through tablet (Group 2 nr. 8); No visual distraction (Group 3 nr. 8). Dental therapy: professional oral hygiene.	Main result: The use of VR glasses was more effective in reducing dental anxiety rates compared to the groups experiencing visual distraction via tablets and no visual distraction. Child-level: The anxiety scores from the baseline and during and after treatment decreased in the VR glasses group; no difference was noted between groups for the pulse rate and blood pressure. Dentist-level: M.D. Care-giver-level: M.D.
Suresh et al., 2019 Clinical study [23]	Nr. of participants: 68 Age: 8–15 years old Target Population: ASD	Aim: To assess the effectiveness of VR in managing children’s dental anxiety and behavior. Assessment tool: Frankl’s rating scale and Vehnham’s picture scale	Intervention: VR eyeglasses (session 1); conventional behavior management techniques (session 2). Dental therapy: Session 1–2: non-invasive dental treatment (professional oral hygiene, dental restorations, topical fluoride application, sealant placement).	Main result: VR can be effective in managing behavior in ASD children during non-invasive dental treatment. Child-level: Anxiety scores with VR eyeglasses decreased but remained high; positive behavior scores increased (from 22% to 55%). Dentist-level: The use of VR may improve the management of children, with no disruption, by the dental team during dental treatment. Care-giver-level: M.D.

Abbreviations: Number—“Nr.”; missing data—“M.D.”; audiovisual—“AV”; virtual reality—“VR”; tell–show–do—“TSD”; Revised Face, Legs, Activity, Cry, Consolability—“r-FLACC”; Revised Faces Pain Scale—“FPS.R”; Facial Image Scale—“FIS”; Visual Analogue Scale—“VAS”, autism spectrum disorder—“ASD”.

## Data Availability

Reported data are available on the MEDLINE/PubMed, Scopus, and Web of Science databases.
